# In person versus remote cognitive rehabilitation in patients with subjective cognitive decline or neurocognitive disorders: what factors drive patient’s preference?

**DOI:** 10.3389/fpsyg.2023.1266314

**Published:** 2023-10-04

**Authors:** Sara Bernini, Elena Ballante, Federico Fassio, Silvia Panzarasa, Silvana Quaglini, Chiara Riccietti, Alfredo Costa, Stefano F. Cappa, Cristina Tassorelli, Tomaso Vecchi, Sara Bottiroli

**Affiliations:** ^1^Dementia Research Center, IRCCS Mondino Foundation, Pavia, Italy; ^2^Department of Political and Social Sciences, University of Pavia, Pavia, Italy; ^3^BioData Science Unit, IRCCS Mondino Foundation, Pavia, Italy; ^4^Department of Public Health, Experimental and Forensic Medicine, Section of Biostatistics and Clinical Epidemiology, University of Pavia, Pavia, Italy; ^5^Department of Electrical, Computer and Biomedical Engineering, University of Pavia, Pavia, Italy; ^6^Imaging Radiology and Interventional Neuroradiology Unit, Department of Neurosurgery, Fondazione IRCCS Istituto Neurologico Carlo Besta, Milan, Italy; ^7^Department of Brain and Behavioral Sciences, University of Pavia, Pavia, Italy; ^8^IUSS Cognitive Neuroscience (ICoN) Center, Scuola Universitaria di Studi Superiori IUSS, Pavia, Italy; ^9^Headache Science and Neurorehabilitation Centre, IRCCS Mondino Foundation, Pavia, Italy; ^10^Cognitive Psychology Research Center, IRCCS Mondino Foundation, Pavia, Italy; ^11^Faculty of Law, Giustino Fortunato University, Benevento, Italy

**Keywords:** neurocognitive disorder, cognitive rehabilitation, telerehabilitation, cognitive reserve, lifestyle

## Abstract

**Background:**

To date, there is still a lack of consensus for identifying the ideal candidate for cognitive telerehabilitation (TR). The main goal of the present study is to identify the factors associated to the preference for either TR or in-person cognitive training (CT) programs in older adults at risk of dementia or with early cognitive impairment.

**Methods:**

A sample of 56 participants with subjective cognitive decline or neurocognitive disorders eligible for CT were enrolled at the Dementia Research Center and Neurorehabilitation Unit of IRCCS Mondino Foundation. All individuals underwent a baseline assessment to capture their complete profile, including cognitive reserve and lifestyle habits, sociodemographic characteristics, cognitive functioning, and mental health. Patients were then asked their preference for TR or in-person CT, before being randomized to either treatment as per protocol procedures. Statistical analyses included explorative descriptive approach, logistic regression, and non-parametric models to explore the overall contribution of each variable.

**Results:**

The two (TR and in-person) preference groups were similar for cognitive functioning and mental health status. Socio-demographic and lifestyle profiles seem to be the most important factors to influence the preference in terms of the area under the curve (AUC) of the models. The two preference groups differed in terms of socio-demographic characteristics (e.g., level of technological skills, age, and distance from the clinic). Furthermore, participants who selected the TR modality of CT had significantly higher levels of cognitive reserve and adopted more protective lifestyle habits (e.g., regular physical activity, Mediterranean diet) when compared to those who preferred in-person CT.

**Discussion:**

These findings highlight that the preference to receive CT delivered by TR or in person is a complex issue and is influenced by a variety of factors, mostly related to lifestyle habits and sociodemographic characteristics. Availability of profiles of patients that may be more attracted to one or the other modality of TR may help promote shared decision-making to enhance patient experience and outcomes.

## 1. Introduction

Dementia or major neurocognitive disorder (MNCD) refers to multi-domain cognitive deficits resulting in a significant interference with independence in everyday functioning (American Psychiatric Association, 2013). The transitional phase between normal aging and dementia is instead defined as Mild Neurocognitive Disorder (mNCD) and it is characterized by both subjective complaints and objective cognitive impairments not interfering with basic activities of daily life (American Psychiatric Association, 2013). Given that pathological changes in MNCD could have occurred years ahead of the manifestation of mNCD (Jessen, 2010), there should be a “pre-mNCD” phase before its manifestation, that is the so called subjective cognitive decline (SCD; Jessen et al., 2014). SCD is indeed an intermediate state between normal cognition and mNCD that may predict the development of objective cognitive decline (Reisberg and Shulman, 2009; Jr et al., 2011). Thus, SCD and mNCD represent critical stages for early diagnosis and intervention of MNCD, also at the light of limited effects of pharmacological therapies on slowing cognitive symptoms (Thoene-Otto et al., 2012). Consequently, non-pharmacological intervention strategies, such as cognitive training (CT), gained increasing attention in these populations (Li et al., 2011; Bahar-Fuchs et al., 2013; Rojas et al., 2013; Andrieu et al., 2015; Smart et al., 2017).

The recent progress of information and communication technologies (ICT) has fostered an increasing interest in the use of technological tools also for CT (Hill et al., 2017). In this field, computer-based CT emerges as a treatment solution with many advantages for both the therapist and the patients, besides overcoming many limitations of traditional interventions (Irazoki et al., 2020). More recently, ICT-based reliable rehabilitation services have been implemented on a large scale, at distance, and directly at home, configuring what is called telerehabilitation (TR) (Zampolini et al., 2008; Cotelli et al., 2019; Maresca et al., 2020). TR can guarantee a continuum of care with the possibility to perform rehabilitation in an ecologic environment, independently or with the help of a caregiver. As a result, TR promotes equitable health care regardless of patients’ geographically distant living areas or disability, potentially reducing the overall cost of care compared with in-person rehabilitation (Peretti et al., 2017). In this field, most scientific efforts have been focused to devise user friendly systems than can be accessed and used without the direct intervention of the therapist (Castilla et al., 2020; De Cola et al., 2020). Another important feature of the ICT-supported TR modalities is the possibility to tailor duration and frequencies of treatment sessions according to patients’ characteristics (Bernini et al., 2021b).

Currently, a growing number of studies have explored the comparability of TR and in-person (in-P) CT in terms of cognitive outcomes (Jelcic et al., 2014; Realdon et al., 2016; Bernini et al., 2021b). There is however a lack of analysis of the characteristics of end users that make them more likely to prefer a TR modality over the other. Elderly generations are indeed familiar with classic technologies, such as television, radio, and telephone, but may be less inclined to use advanced ICT modalities (Fadzil et al., 2022). Beside the diagnostic category of patients, it would be important for clinicians to consider what are the factors underlying the interest in receiving this technological service, inclination to use the program, biases and expectations toward different program options, before offering digital CT solutions (e.g., TR, in-P CT). For instance, it has been shown in the field of Parkinson’s Disease (PD) that some socio-demographic variables, such as age and education, could be moderator of differences in the efficacy of an in-P CT (Sanchez-Luengos et al., 2021). Also cognitive functioning prior to receive CT may predict the outcome of the intervention: lower cognitive profiles resulted associated to more marked (but short-lasting) responsiveness to a computer-based CT (Bernini et al., 2023). Moreover, an active lifestyle may be a determining factor in cognitive rehabilitation given that it can help in increasing attendance and participation in sessions and achieving positive results, as demonstrated in older adults at risk of dementia receiving CT (Küster et al., 2016). Unfortunately, a consensus about the ideal candidate for either type of CT is still lacking (Di Tella et al., 2021) and, to the best of our knowledge, no study has been devoted to investigate what and how individual factors could influence patients’ preference for receiving CT (Isernia et al., 2019). Thus, further investigation on these topics is needed.

In the last years, our group has devised and implemented a cognitive rehabilitation (CoRe) software for an in-person computer-based CT (Alloni et al., 2017, 2018). CoRe was effective in restoring lost brain function and slowing degenerative diseases in early cognitive decline, compared with traditional interventions (Bernini et al., 2019, 2021a, 2023; Rodella et al., 2021). With a view to starting/continuing the CoRe program remotely (Quaglini et al., 2019), we have recently developed a “home” version (called HomeCoRe), able to provide a cognitive intervention directly at home (Bernini et al., 2021b,c).

In the present study, we explored the aspects associated to the preference for CT delivered with the HomeCoRe system (TR) or with the CoRe tool (in-P) for CT in patients with SCD or NCD. We focused on four profiles: lifestyle, socio-demographic, cognitive, and mental-health. In particular, we expected lifestyle to emerge as relevant among the others. This consideration takes its origin from the assumption that the adoption of protective behaviors in everyday life (e.g., cognitive engagement, leisure activities, nutrition, physical activity, health status, etc.) would make patients more open to new and emerging technological opportunities offered to support and facilitate successful aging (Dogra et al., 2022).

## 2. Materials and methods

### 2.1. Study design and participants

The data were collected within a larger ongoing randomized controlled trial (Bernini et al., 2021b) where we compared TR delivered with HomeCoRe vs. in-P CT delivered with CoRe in subjects with SCD or NCD. All subjects gave their informed consent to collect the data analyzed in this report. The study was conducted in accordance with the Declaration of Helsinki, and the protocol approved by the Ethics Committee (San Matteo Hospital, Pavia, Italy - # P-20210032883; # 2022161/22).

Participants were recruited (January 2022 – January 2023) from the Dementia Research Center outpatient service and Neurorehabilitation Unit of IRCCS Mondino Foundation of Pavia and screened for eligibility criteria through a clinician evaluation by both an experienced neurologist and neuropsychologist.

The inclusion criteria for participants were: (a) age > 50 years, (b) education >5 years; (c) a diagnosis of SCD (Jessen et al., 2014), mNCD, and MNCD (American Psychiatric Association, 2013) due to Alzheimer’s disease or Vascular dementia; (d) Clinical Dementia Rating (CDR) (Hughes et al., 1982) score ranging between 0 and 1; (e) Mini-Mental State Examination (MMSE; Measso et al., 1993) raw score ≥ 20.

The exclusion criteria were: (a) presence of cognitive impairment secondary to an acute or general medical disorder (e.g., brain trauma or tumor), (b) presence of severe neuropsychiatric conditions (e.g., mood and behavioral disorders), (c) presence of severe sensory disorder (e.g., deafness or blindness) or motor functioning deficits in dominant upper limb.

Fifty-six participants were considered eligible for inclusion and underwent a baseline assessment (T0) to collect sociodemographic data, clinical and neuropsychological measures, and their intervention preference. Information about participants profiling in the considered domains were collected through self-report questionnaires and specific standardized instruments.

The dataset used for this study was shared on Zenodo platform in accordance with the guidelines of GDPR.

### 2.2. Socio-demographic profile

An anamnestic interview was carried out in order to collect socio-demographic information, such as age, education, marital and parenting status, possible family member availability for supporting TR, past and present profession, income, and distance from the clinic. An *ad-hoc* self-report assessment of Technology Skills (TS) was also performed in which participants were asked to assess their level of familiarity with computers on a Likert scale with 0 (none), 1 (poor), 2 (modest), 3 (good), and 4 (excellent) as possible responses.

### 2.3. Lifestyle profile

#### 2.3.1. Cognitive reserve index questionnaire

It estimates cognitive reserve by means of a collection of participant-related factors (Nucci et al., 2012). It returns a total score and three sub dimension-related scores, as reported below. Higher scores are indicative of higher cognitive reserve. The Cognitive Reserve Index questionnaire (CRIq) provides three subscores:

1. CRI-education refers to the degree of schooling attained by an individual during the life span
2. CRI-working activity records the type and number of years of paid employment held by an individual. Different levels of work employment have been identified that differ in the cognitive commitment required as well as the level of responsibility assumed
3. CRI-leisure time refers to all those activities that are usually performed outside the hours of work or school attendance.

#### 2.3.2. Lifestyle for brain health (LIBRA)

It is a questionnaire that can help identify and monitor lifestyle risk/protection of dementia by targeting modifiable risk factors (MRF) (Schiepers et al., 2018; Franchini et al., 2019). The score ranges from −5.9 to +12.7. Higher scores correlate with higher risk of dementia and cognitive impairment. It investigates the presence or absence of each of the following MRF evaluated thorough a semi-structured interview: (1) coronary heart disease, (2) diabetes, (3) hypercholesterolemia, (4) hypertension, (5) depression, (6) obesity, (7) smoking, (8) alcohol intake, (9) physical activity, (10) cognitive activity, (11) Mediterranean diet, (12) renal dysfunction. According to the presence or absence of each MRF, a specific score has been assigned that concurred to the determination of the LIBRA index, as explained in the Italian validation of this instrument (Franchini et al., 2019). In addition to consider the global index, we also treated each MRF as present or absent, dichotomously.

### 2.4. Cognitive profile

We used the following standardized tests to assess five cognitive domains:

- Global cognitive functioning: mini-mental state examination (MMSE) (Measso et al., 1993) and Montreal cognitive assessment (MoCA) (Conti et al., 2015)
- Episodic long-term memory: logical memory test (Novelli et al., 1986; Spinnler, 1987), Rey’s 15 words test immediate-delayed recall (Carlesimo et al., 1996), Rey complex figure delayed recall (Caffarra et al., 2002)
- Logical-executive functions: Raven’s Matrices 1947 (Carlesimo et al., 1996), Frontal Assessment Battery (FAB) (Appollonio et al., 2005); semantic (Novelli et al., 1986) and phonological fluencies (FAS) (Carlesimo et al., 1996), Rey complex figure copy (Caffarra et al., 2002)
- Working memory: verbal span, digit span, Corsi’s block-tapping test span (Spinnler, 1987)
- Attention/processing speed: attentive matrices (Spinnler, 1987), Trail Making Test (TMT) (Giovagnoli et al., 1996).

The raw scores for each neuropsychological test underwent adjustments for age, sex, and education, and were subsequently compared to the reference values for the Italian population. Following this comparison, the adjusted scores were converted into equivalent scores (Capitani and Laiacona, 1997). The average of the equivalent test scores reported for each domain was then calculated.

Patients’ diagnosis (i.e., SCD, mNCD, and MNCD) was considered as part of the cognitive profile as well.

### 2.5. Mental health profile

#### 2.5.1. Beck depression inventory

Beck depression inventory (BDI) (Beck et al., 1996) for depressive symptoms. It consists of 21 items that investigate the severity of depressive symptoms. For each set of statements, the subject is asked to choose the one that best describes his/her current situation. The total score is calculated as the sum of the scores of the individual items. A cut off of 9 was considered.

#### 2.5.2. 36-Item short form health survey

36-Item short form health survey (SF-36) (Apolone and Mosconi, 1998) assesses health-related quality of life. It is composed of 36 Likert scale items that return a score related eight sub-scales: (1) physical functioning, (2) role limitations (physical), (3) role limitations (emotional), (4) energy/vitality, (5) mental health, (6) social functioning, (7) bodily pain, and (8) general health perceptions. High scores are indicative of better perceived health status.

### 2.6. TR and in-P CT programs

Both the HomeCoRe and the CoRe are research software tools developed within a long-lasting collaboration between clinicians from the IRCCS Mondino and bioengineers from the University of Pavia. Both tools allow a participant-tailored intervention aimed at stimulating several cognitive abilities through a series of sessions of exercises. Participants were informed that they could receive a cognitive intervention consisting of a 6-week program (3 sessions/week, each lasting approximately 45 min). It was further explained that the intervention could be carried out in two different modes: (1) TR, i.e., performed independently by patients at home on a laptop computer provided by the clinic and supervised remotely, or (2) in-P, i.e., in the hospital setting, on a desktop PC located in the clinic and supervised by the therapist.

### 2.7. Statistical analysis

In the present study, we compared subjects who preferred in-P rehabilitation with CoRe versus subjects who expressed their preference for TR with HomeCoRe by explorative descriptive analysis. Since the majority of variable did not respect the normality assumption at Shapiro–Wilk test and Q-Q plot, we decided to use a non-parametric test to be conservative. Mann–Whitney test was used for continuous variables while the Chi-square test for categorical ones with Yates’ continuity correction (or Fisher’s test if ≥25% of the expected frequencies were less than *n* = 5). Logistic regression models were implemented for the 4 areas of interest. Starting from the full model for each area, a stepwise selection method of the variables was used identifying the lowest Akaike information criterion (AIC) value to obtain the best model for each area. Area Under the Curve (AUC) and accuracy were assessed for each logistic model in order to identify the most explanatory areas. In these models, total/total weighted scores (CRIq global score, LIBRA index, global cognition functioning) were not considered to avoid collinearity, since the single items were included. To investigate any variation of significance between univariate and multivariate analysis, association between selected variables was explored through Spearman’s rank correlation or Mann–Whitney/Kruskal–Wallis test. To confirm regression model results, a non-parametric model (Random Forest model: RF) was used to explore the overall contribution of each variable. In this model, all the variables available were considered. The number of trees for each model was set considering the lowest out-of-bag error. The fit of the model was assessed by confusion matrix accuracy and AUC value. VIMP (Variable IMPortance) values were used to identify the importance of each variable according to our outcome. Overall, the significance was set as a value of p lower than 0.05. Analysis were performed in R environment (v. 4.2.3), using “stats” and “randomForest” packages for stepwise models selection and RF.

Continuous variables are reported as medians and 25th–75th percentiles. Categorical variables are described as frequencies and percentages.

A minimum sample of 52 subjects was considered enough to identify an AUC value of 0.80 of a statistical model, assuming a proportion of sample choosing presence or at home equal to 0.5, an alpha value of 0.05 and a total width of confidence interval equal to 0.25.

## 3. Results

### 3.1. Socio-demographic profile

Descriptive statistics for socio-demographic profiles as a function of preference group are reported in Table 1. The TR group was significantly younger (*p*_M–W_ = 0.003), more technologically skilled (*p*_Fisher_ = 0.002), and lived farther from the clinic than the in-P group (*p*_M–W_ = 0.003). The two preference groups were instead similar in terms of sex, education, family member availability, marital and parental status, past and actual profession, and income.

**Table 1:** Socio-demographic characteristics of the two groups of subjects (expressed as median and 25th–75th percentiles or as absolute value and percentage).

	Telerehabilitation (*N* = 31)	In-person rehabilitation (*N* = 25)	Value of *p*
**Age**			
Median [Q1, Q3]	71.0 [64.5, 75.0]	75.0 [73.0, 77.0]	0.003
**Gender**			
Male	14 (45.2%)	12 (48.0%)	0.90
Female	17 (54.8%)	13 (52.0%)	
**Years of education**			
Median [Q1, Q3]	11.0 [8.00, 14.0]	8.00 [8.00, 11.0]	0.076
**Technological skills**			
None	2 (6.5%)	11 (44.0%)	0.002
Poor	3 (9.7%)	5 (20.0%)	
Modest	15 (48.4%)	7 (28.0%)	
Good	10 (32.3%)	2 (8.0%)	
Excellent	1 (3.2%)	0 (0%)	
**Family member**			
Absence	7 (22.6%)	7 (28.0%)	0.877
Presence	24 (77.4%)	18 (72.0%)	
**Marital status**			
Married/cohabitant	26 (83.9%)	18 (72.0%)	0.454
Widowed	5 (16.1%)	7 (28.0%)	
**Offspring**			
No	3 (9.7%)	2 (8.0%)	0.90
Yes	28 (90.3%)	23 (92.0%)	
**Current occupation**			
Retired	26 (83.9%)	25 (100%)	0.058
Working	5 (16.1%)	0 (0%)	
**Past occupation**			
Unskilled worker	0 (0%)	4 (16.0%)	0.121
Craftsman or skilled worker	9 (29.0%)	8 (32.0%)	
Concept clerk	14 (45.2%)	8 (32.0%)	
Small/large company manager	8 (25.8%)	5 (20.0%)	
**Income**			
Low	9 (29.0%)	12 (48.0%)	0.343
Medium	14 (45.2%)	8 (32.0%)	
High	8 (25.8%)	5 (20.0%)	
**Distance from the hospital (km)**			
Median [Q1, Q3]	20.0 [9.00, 31.5]	6.00 [4.00, 20.0]	0.003

The stepwise logistic models (Table 2) highlighted how the most important variables in this area were the distance in kilometres from the clinic (*p* = 0.017), the age of the subject and the level of TS (statistical significance for modest and good skills vs. none: *p* = 0.015 and *p* = 0.012, respectively). Gender and the presence of children features were kept by stepwise regression, but without statistical significance. In the logistic model above, the unitary increase in distance from the clinic (km) was associated with a 10% reduction in the odds for the “face-to-face” approach preference. Overall, the socio-demographic area had an excellent explanatory power on the preference with an AUC of 0.88 and an accuracy of 0.80. No association emerged from age and distance from the clinic, while there was some difference among TS groups for age (*p*_K–W_ < 0.001). Statistical test showed no difference between distance from the clinic and TS groups.

**Table 2:** Logistic regression model for predicting preference by considering socio-demographic profile.

Characteristic	OR	95% CI	Value of *p*
Age	1.12	0.97, 1.35	0.157
**Gender**			
Male	Ref.	Ref.	
Female	0.23	0.03, 1.20	0.104
**Technological Skills**			
None	Ref.	Ref.	
Poor	0.30	0.02, 3.89	0.358
Modest	0.05	0.00, 0.42	0.015
Good	0.01	0.00, 0.22	0.012
Excellent	0.00	-	0.993
**Offspring**			
No	Ref.	Ref.	
Yes	0.07	0.00, 2.74	0.169
Distance from the hospital (km)	0.90	0.82, 0.97	0.017

AUC: 0.88; Accuracy: 0.80. OR, odds ratio; CI, confidence interval; Ref, reference category.

### 3.2. Lifestyle profile

Descriptive statistics for lifestyle profiles as a function of preference group are reported in Table 3. The TR group had a significantly higher total score in the CRIq (*p*_M–W_ = 0.031) and in the CRIq leisure time score (*p*_M–W_ = 0.015). The two groups were similar in terms of CRIq working activity and CRIq education scores. As the LIBRA index, the TR group scored significantly lower that the in-P group (*p*_M–W_ = 0.031). Moreover, the TR group included a significantly higher prevalence of individuals physically active (*p*_Chi2_ = 0.035) and following the Mediterranean diet (*p*_Chi2_ = 0.017). No other differences between both groups were found in the other MRFs.

**Table 3:** Lifestyle characteristics of the two groups of subjects (expressed as median and 25th–75th percentiles or as absolute value and percentage).

	Telerehabilitation (*N* = 31)	In-person rehabilitation (*N* = 25)	Value of *p*
**CRIq global score**			
Median [Q1, Q3]	107 [97.0, 116]	96.0 [86.0, 109]	0.031
**CRIq education score**			
Median [Q1, Q3]	105 [95.0, 113]	101 [96.0, 108]	0.804
**CRIq working activity score**			
Median [Q1, Q3]	108 [99.0, 121]	100 [87.0, 110]	0.093
**CRIq leisure time score**			
Median [Q1, Q3]	100 [88.0, 108]	89.0 [78.0, 100]	0.015
**Coronary heart disease MRF**			
No	28 (90.3%)	19 (76.0%)	0.272
Yes	3 (9.7%)	6 (24.0%)	
**Diabetes MRF**			
No	26 (83.9%)	22 (88.0%)	0.713
Yes	5 (16.1%)	3 (12.0%)	
**Hypercholesterolemia MRF**			
No	20 (64.5%)	15 (60.0%)	0.945
Yes	11 (35.5%)	10 (40.0%)	
**Hypertension MRF**			
No	20 (64.5%)	16 (64.0%)	0.90
Yes	11 (35.5%)	9 (36.0%)	
**Obesity MRF**			
No	27 (87.1%)	23 (92.0%)	0.682
Yes	4 (12.9%)	2 (8.0%)	
**Smoking MRF**			
No	27 (87.1%)	24 (96.0%)	0.367
Yes	4 (12.9%)	1 (4.0%)	
**Physical activity MRF**			
No	20 (64.5%)	23 (92.0%)	0.035
Yes	11 (35.5%)	2 (8.0%)	
**Cognitively activity MRF**			
No	18 (58.1%)	19 (76.0%)	0.260
Yes	13 (41.9%)	6 (24.0%)	
**Mediterranean diet MRF**			
No	14 (45.2%)	20 (80.0%)	0.017
Yes	17 (54.8%)	5 (20.0%)	
**Renal dysfunction MRF**			
No	30 (96.8%)	23 (92.0%)	0.581
Yes	1 (3.2%)	2 (8.0%)	
**LIBRA index**			
Median [Q1, Q3]	0.100 [−1.45, 1.75]	2.10 [0.100, 2.70]	0.031

CRIq, cognitive reserve index questionnaire; MRF, modifiable risk factors; LIBRA, lifestyle for brain health.

Regarding the lifestyle area, the stepwise approach for the logistic model regression (Table 4) selected the CRIq for free time, smoking habits, and the Mediterranean diet, even if they were not significant. Overall, the lifestyle area had a good explanatory power on the preference with an AUC of 0.75 and an accuracy of 0.75. To explain the loss of significance of the selected variables, we observed that people following a Mediterranean diet had a higher CRIq leisure time score (105 *vs* 88; *p*_M–W_ < 0.001), while no significant difference emerged between smokers and no-smokers. There was no association between smoking habits and diet.

**Table 4:** Logistic regression model for predicting preference by considering lifestyle profile.

Characteristic	OR	95% CI	Value of *p*
CRIq leisure time score	0.97	0.92, 1.01	0.163
**Smoking MRF**			
No	Ref.	Ref.	
Yes	0.20	0.01, 1.56	0.171
**Mediterranean diet MRF**			
No	Ref.	Ref.	
Yes	0.28	0.07, 1.05	0.065

AUC: 0.75; Accuracy: 0.75. OR, odds ratio; CI, confidence interval; Ref., reference category; CRIq, cognitive reserve index questionnaire; MRF, modifiable risk factors.

### 3.3. Cognitive profile

Descriptive statistics for cognitive profiles at enrollment as a function of preference group are reported in Table 5. The two preference groups did not differ in any of the cognitive domains assessed (i.e., global cognitive functioning, episodic long-term memory, logical-executive functions, working memory, and attention/ processing speed) neither in terms of prevalence of diagnostic category.

**Table 5:** Cognitive characteristics of the two groups of subjects (expressed as median and 25th–75th percentiles or as absolute value and percentage).

	Telerehabilitation (*N* = 31)	In-person rehabilitation (*N* = 25)	Value of *p*
**Diagnostic category**			
Subjective cognitive decline	6 (19.4%)	3 (12.0%)	0.662
Mild neurocognitive disorder	23 (74.2%)	19 (76.0%)	
Major neurocognitive disorder	2 (6.5%)	3 (12.0%)	
Mini Mental State Examination*			
Median [Q1, Q3]	1.00 [1.00, 1.00]	1.00 [1.00, 1.00]	0.490
Montreal Cognitive Assessment*			
Median [Q1, Q3]	2.00 [0.500, 3.00]	2.00 [1.00, 3.00]	0.643
Global cognitive functioning*			
Median [Q1, Q3]	1.50 [0.500, 2.00]	1.50 [1.00, 2.00]	0.749
Attention/processing speed*			
Median [Q1, Q3]	2.33 [1.33, 3.33]	2.00 [1.33, 2.67]	0.226
Episodic long-term memory*			
Median [Q1, Q3]	1.50 [0.750, 2.38]	2.00 [1.25, 2.75]	0.220
Logical-executive functions*			
Median [Q1, Q3]	3.00 [2.30, 3.40]	2.40 [2.00, 3.00]	0.155
Working memory*			
Median [Q1, Q3]	2.67 [2.33, 3.00]	2.67 [1.67, 3.00]	0.764

^*^Indicates performance expressed in equivalent scores.

The stepwise logistic model (Table 6) selected attention processing speed and episodic long-term memory, both without statistical significance. Overall, the cognitive profile had a poor explanatory power on the preference with an AUC of 0.65 and an accuracy of 0.59.

**Table 6:** Logistic regression model for predicting preference by considering cognitive profile.

Characteristic	OR	95% CI	Value of *p*
Attention/processing speed	0.64	0.36, 1.06	0.097
Episodic long-term memory	1.63	0.91, 3.03	0.108

AUC: 0.65; Accuracy: 0.59. OR, odds ratio; CI, confidence interval.

### 3.4. Mental health profile

Descriptive statistics for mental health profiles as a function of preference group are reported in Table 7. The TR group had a significantly higher physical functioning QoL than the in-P group (*p* = 0.037). The two groups did not differ in any of the other SF-36 domains neither in the BDI score.

**Table 7:** Mental health characteristics of the two groups of subjects (expressed as median and 25th–75th percentiles).

	Telerehabilitation (*N* = 31)	In-person rehabilitation (*N* = 25)	Value of *p*
**Depressive symptoms**			
Median [Q1, Q3]	8.00 [5.00, 15.0]	8.00 [5.00, 11.0]	0.503
**Physical functioning**			
Median [Q1, Q3]	90.0 [72.5, 95.0]	75.0 [55.0, 90.0]	0.037
**Role limitations (physical)**			
Median [Q1, Q3]	75.0 [61.0, 100]	100 [50.0, 100]	0.90
**Bodily pain**			
Median [Q1, Q3]	70.0 [45.0, 100]	67.5 [45.0, 90.0]	0.782
**Social functioning**			
Median [Q1, Q3]	75.0 [62.5, 87.5]	75.0 [62.5, 87.5]	0.827
**Mental health**			
Median [Q1, Q3]	64.0 [56.0, 70.5]	64.0 [60.0, 72.0]	0.280
**Role limitations (emotional)**			
Median [Q1, Q3]	66.7 [33.3, 100]	66.7 [33.3, 100]	0.875
**Energy/vitality**			
Median [Q1, Q3]	55.0 [50.0, 67.5]	55.0 [50.0, 60.0]	0.371
**General health perceptions**			
Median [Q1, Q3]	50.0 [45.0, 60.0]	55.0 [45.0, 65.0]	0.460
**Health changes**			
Median [Q1, Q3]	50.0 [50.0, 50.0]	50.0 [25.0, 50.0]	0.300

The stepwise logistic model (Table 8) selected the SF-36 physical functioning subscale variable (*p* = 0.050). Overall, the cognitive profile had a poor explanatory power on the preference with an AUC of 0.66 and an accuracy of 0.57.

**Table 8:** Logistic regression model for predicting preference by considering mental-health profile.

Characteristic	OR	95% CI	Value of *p*
Physical functioning	0.97	0.94, 1.00	0.050

AUC: 0.66; Accuracy: 0.57. OR, odds ratio; CI, confidence interval.

### 3.5. Random forest

**Figure 1:**
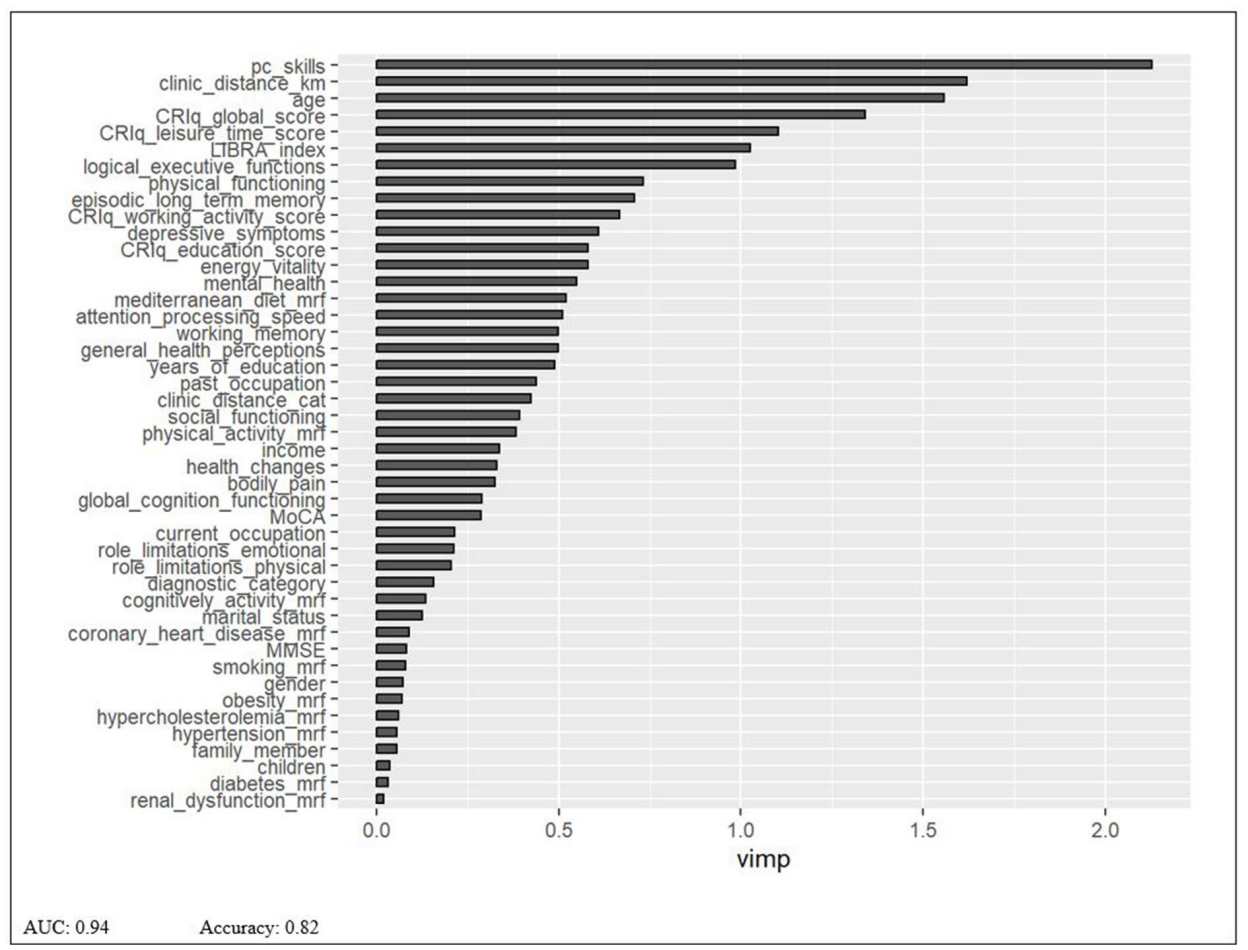
Random forest model.

The RF model was implemented on 400 decision trees, considering 9 variables at each split. The first seven variables identified as the most important to classify subjects into the TR versus the in-P group are the following: Technological Skills, distance from the clinic, age, CRIq global score, CRIq leisure time score, the LIBRA index, logical/executive functioning (see Figure 1).

## 4. Discussion

The present study aimed to explore the characteristics of patients at risk of dementia or with early cognitive impairment that are associated to preference for the TR or the in-P modality of CT. We hypothesized that patients’ lifestyle profile could be the main factor driving the preference, given that an active lifestyle may render patients more open to new and emerging technological opportunities offered to support and facilitate aging (Dogra et al., 2022). Our findings seem to support this hypothesis. In fact, the best performances of the logistic regression models were obtained in the sociodemographic and lifestyle areas, compared to mental health and cognitive.

Participants preferring TR were characterized by higher levels of cognitive reserve, in particular in what concerns leisure cognitively demanding activities carried out during the entire life span. Engaging in cognitive, social, and physical activities throughout life is known to contribute to high levels of cognitive reserve, which protects individuals from sequelae of neural damage, reduces the risk of developing dementia, and slows the rate of cognitive decline due to normal aging (Stern, 2009; Clare et al., 2017). Hence, high levels of cognitive reserve can prevent cognitive decline and stimulate intellectual curiosity and may potentially contribute to increase subjects’ openness to use innovative technological solutions, such as HomeCore-delivered TR in our case, which would not only simplify their daily activities, but also help them lead a more active life (Ranieri et al., 2021). A very recent consensus work (Stern et al., 2023) considered cognitive reserve as influenced by multiple genetic and environmental factors operating at various points or continuously across the lifespan. For example, proxies for cognitive reserve in human studies have included characteristics associated with both endowment and experience, including Intelligence Quotient at an early age, exposures to cognitive stimuli across the age span, education, occupational exposures, leisure activities, social networks, and other factors. In the context of the present study, we measured this construct through the CRIq, according to those considering education, occupation and leisure activities as the most frequently used proxies of cognitive reserve (Valenzuela and Sachdev, 2007; Solé-Padullés et al., 2009; Jones et al., 2010). Future studies should explore this topic further using more comprehensive proxies.

Moreover, we also found that participants who preferred HomeCoRe had lower LIBRA index, suggesting that they were more prone to adopt a “protective” lifestyle that may reduce their individual risk of dementia in later life (Linardakis et al., 2013; Schiepers et al., 2018). Specifically, an increase in the global LIBRA score by one point is related to a 19% higher risk for developing dementia (Schiepers et al., 2018). It is important to consider that cognitive reserve is not a static construct but rather it evolves throughout the entire life course (Valenzuela and Sachdev, 2006), and lifestyle habits can help protect older people from cognitive decline and dementia by supporting the development, connectivity, and maintenance of brain networks (Clare et al., 2017). In this regard, the results of the present study suggest that greater cognitive reserve and adoption of healthy lifestyles characterize those individuals more likely to adopt new technologies (Kalache and Gatti, 2003). Actually, the regression model for the lifestyle area kept variable associated with a healthy behavior, with an overall good performance. The association between Mediterranean diet and CRIq leisure time score was a corroboration of this.

By contrast, participants preferring in-P CT were characterized by age-stereotypic attitudes (Felt et al., 2016), being less interested in adopting new technologies. If we consider that technologies are becoming even more integrated into everyday life, people with less openness to innovation are more likely to become more disenfranchised and disadvantaged (Czaja et al., 2006) and are therefore more at risk to enter a vicious cycle that may negatively influence their cognitive abilities. Other factors were associated with the preference for TR or in-P CT. In particular, we found that the two preference groups differed in some variables, such as age, technological skills, and the distance from the clinic. These findings are not particularly surprising given that these factors are known to influence technology adoption (Czaja et al., 2006; Guzman-Parra et al., 2020; Dequanter et al., 2022). The logistic model for the socio-demographic area was the best model among all. Actually, the RF global model returned socio-demographic and lifestyle features as the most important. The lack of statistical significance in logistic model for some selected features (see age or Mediterranean diet) was to be intended as they were however important in predicting people preference for the rehabilitation delivery modality, either as confounders or as explanatory variables.

Contrary to expectations, cognitive profiles as well as diagnostic categories were not associated to participants’ preferences. This observation seems to be broadly confirmed both by the RF model and by the better performance (in terms of AUC) of the logistic models for each area. We are aware that cognitive abilities such as memory and speed of processing are important to successful performance of technology-based tasks (Charness et al., 2001; Czaja et al., 2001; Sharit et al., 2003) and represent important predictor of computer use (Umemuro, 2004) in the field of normal aging. It should be noted that our study has been carried out on participants at risk of dementia or with early cognitive impairment. One could expect that TR should be perceived as more cognitively demanding, being performed without the therapist support. If we match this result with the different lifestyle profile characterizing the two groups, with TR participants more inclined to active habits in daily life, it could be suggested that other factors instead of cognitive profile may be driving this preference. We also did not find any impact of the mental health profile on participants’ preference for TR vs. in-P CT. Again, others (Guzman-Parra et al., 2020) have shown that depression and health status were associated to a better attitude toward new technologies in early stages of dementia. However, our findings can be related to the fact that our sample of older adults was relatively mentally healthy and they had a fairly stable mood. Future studies with a larger sample might further shed light on the role of cognitive and mental health factors in determining participants’ preferences for technologies.

Overall, our findings suggest that the preference to receive CT delivered by TR or in person is a complex issue influenced by a variety of factors, mostly related to lifestyle habits and sociodemographic characteristics. The relationships among these variables are complex, indicating that people’s choices about preferring a particular CT cannot be explained solely by their clinical condition, including their diagnostic category and cognitive functioning. Several studies have shown that attitudes toward technology, in our case toward CT, are an important predictor of the acceptance of these tools (Al-Gahtani and King, 1999; Kelley et al., 1999; Czaja et al., 2006).

## 5. Conclusion

The present study aimed to identify, among each defined areas, the factors most likely to influence the preference in CT in delivery modalities. Based on our results, we believe that clinicians, when recommending cognitive rehabilitation to patients at risk for dementia, should not only consider their level of impairment, but also sociodemographic (i.e., level of technological skills, age, and distance from the clinic) and lifestyle items (i.e., cognitive reserve, regular physical activity, Mediterranean diet), because they could influence their preference and thus acceptance.

Understanding additional factors influencing preference and acceptance for a particular deliver modality is crucial to select the most suitable candidates for each intervention modality, as this can potentially influence treatment adherence and success.

## Data availability statement

The datasets presented in this study can be found in online repositories. The names of the repository/repositories and accession number(s) can be found below: https://zenodo.org/record/8159236.

## Ethics statement

The studies involving humans were approved by San Matteo Hospital ethics committee. The studies were conducted in accordance with the local legislation and institutional requirements. The participants provided their written informed consent to participate in this study.

## Author contributions

SBe: Conceptualization, Investigation, Methodology, Writing – original draft. EB: Formal analysis, Data curation, Writing – review & editing. FF: Formal analysis, Data curation, Writing – review & editing. SP: Software, Writing – review & editing. SQ: Software, Writing – review & editing. CR: Data curation, Writing – original draft. AC: Resources, Writing – review & editing. SC: Funding acquisition, Resources, Supervision, Writing – review & editing. CT: Supervision, Writing – review & editing. TV: Supervision, Writing – review & editing. SBo: Conceptualization, Investigation, Methodology, Writing – original draft.
